# Century-long cod otolith biochronology reveals individual growth plasticity in response to temperature

**DOI:** 10.1038/s41598-020-73652-6

**Published:** 2020-10-07

**Authors:** Szymon Smoliński, Julie Deplanque-Lasserre, Einar Hjörleifsson, Audrey J. Geffen, Jane A. Godiksen, Steven E. Campana

**Affiliations:** 1 Institute of Marine Research, P.O. Box 1870, 5817 Nordnes, Bergen, Norway; 2 Faculty of Life and Environmental Sciences, University of Iceland, Reykjavík, Iceland; 3 Marine and Freshwater Research Institute, Reykjavík, Iceland; 4 Department of Biological Sciences, University of Bergen (UiB), Bergen, Norway

**Keywords:** Ecology, Climate-change ecology, Marine biology

## Abstract

Otolith biochronologies combine growth records from individual fish to produce long-term growth sequences, which can help to disentangle individual from population-level responses to environmental variability. This study assessed individual thermal plasticity of Atlantic cod (*Gadus morhua*) growth in Icelandic waters based on measurements of otolith increments. We applied linear mixed-effects models and developed a century-long growth biochronology (1908–2014). We demonstrated interannual and cohort-specific changes in the growth of Icelandic cod over the last century which were mainly driven by temperature variation. Temperature had contrasting relationships with growth—positive for the fish during the youngest ages and negative during the oldest ages. We decomposed the effects of temperature on growth observed at the population level into within-individual effects and among‐individual effects and detected significant individual variation in the thermal plasticity of growth. Variance in the individual plasticity differed across cohorts and may be related to the mean environmental conditions experienced by the group. Our results underscore the complexity of the relationships between climatic conditions and the growth of fish at both the population and individual level, and highlight the need to distinguish between average population responses and growth plasticity of the individuals for accurate growth predictions.

## Introduction

Increasing ocean temperature is considered one of the most important climatic factors that influence biological processes in marine systems, including the dynamics of fish stocks^1^. Many retrospective studies on natural populations have shown a relationship between temperature and individual parameters of fish, such as body growth and body size^2^. These fundamental biological characteristics affect many ecological properties of the individuals often reflected in population metrics, e.g., size at maturation, fecundity, recruitment, or population biomass^3^. In consequence, global warming has significant impacts on fish productivity^4^. Predictions of future climatic impacts on fish populations require a thorough assessment of fish growth-temperature relationships^5^.

**Figure 1:**
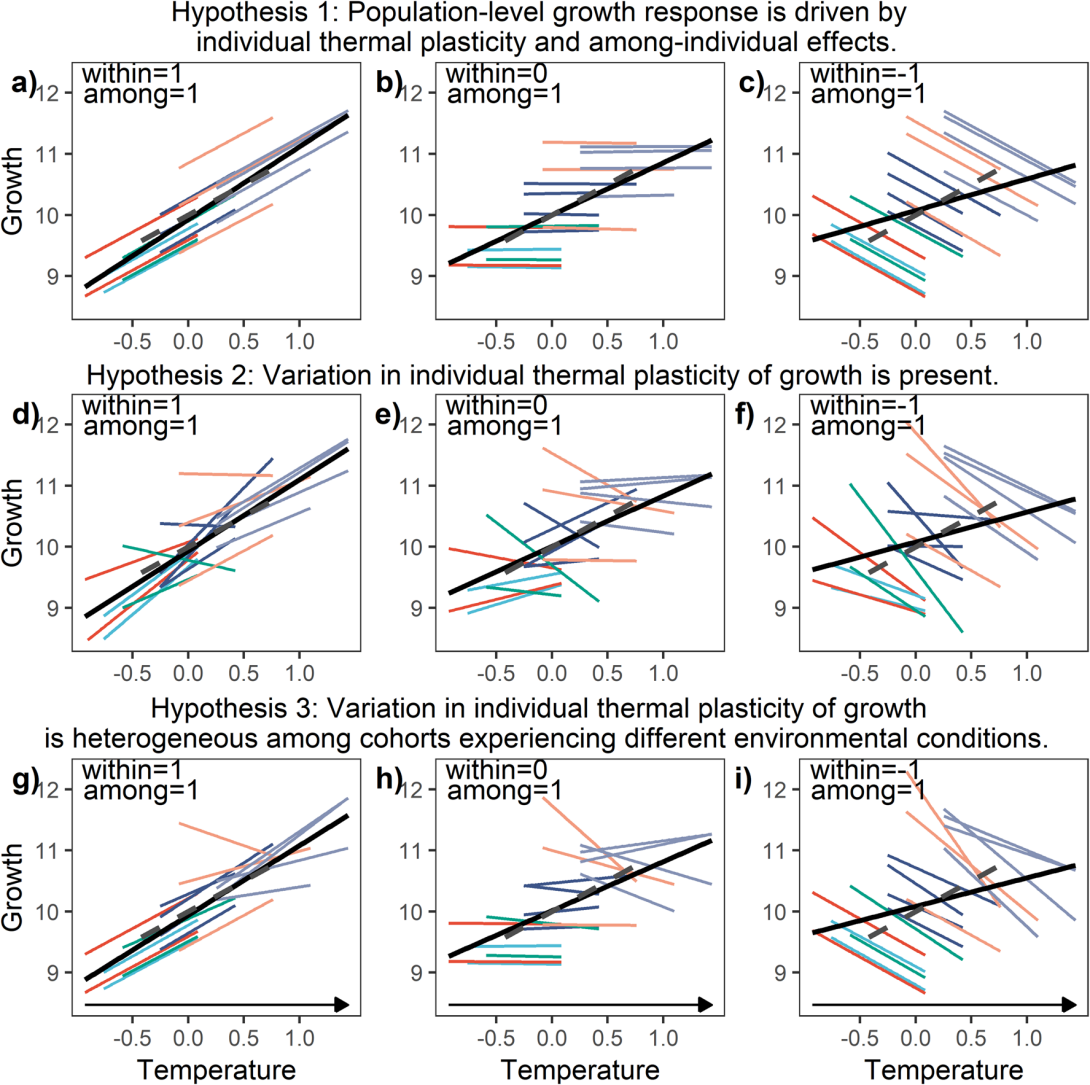
Schematic illustration of different levels of variability in individual thermal plasticity of growth. Colors indicate fish cohorts, colored lines show individual thermal reaction norms, grey dashed lines show among-individual effects, and black solid lines population-level growth response. Example scenarios of within-individual effects (individual plasticity) and among-individual effects of temperature are presented in columns (slopes of -1, 0, or 1 for within-individual and among-individual effects are indicated in the upper-left corners). (**a**–**c**) No significant variation in individual thermal plasticity of growth, (**d**)–(**f**) significant variation in individual plasticity, and (**g)**–(**i**) heterogeneous variance in individual plasticity among the cohorts. Arrows indicate the increasing variance of individual plasticity along the temperature gradient.

Investigations of an individual organism’s responses to temperature change, in addition to the studies of the population-level responses, have become critical for our understanding of the global climate change impacts^6^. Traditionally, in fishery science, the mean size of individuals from a given cohort at successive ages is compared to obtain the mean population growth rate^7^. Typically studies assess the correlation between these annual means for a given trait (e.g. mean population growth rate) with environmental conditions^8^. But the observed population-level responses to the environmental change may be caused both by the individual responses and by between-individual effects associated with the differences in the average environmental conditions experienced by the fish^9^ (Fig. 1a–c). Therefore, the nature of individual responses are difficult to infer from population-level analyses^10^ which can smooth individual expressions of phenotypic traits (e.g., Fig. 1c). Without access to individual growth records, the population-level analyses obscure how individuals track the changing environment^11^.

Otolith biochronologies link growth records from individual fish to produce high-quality, long-term growth sequences^12^. These long-term growth sequences can then be analyzed to disentangle individual effects from population-level responses. Otoliths are calcified structures, part of the acoustic-lateralis system in fish, located in the inner ear. They are composed of calcium carbonate on a protein matrix and grow continuously throughout the life of the fish^13^. Seasonal growth patterns, reflected in translucent and opaque zones, form annual growth increments, similar to tree-rings^14^. Otolith growth is proportional to somatic growth and annual growth increment widths can be measured as a proxy of individual growth to reconstruct individual growth histories^12^. Otolith biochronologies can fill gaps or extend the information on fish growth before the periods in which historical measurements of fish body size-at-age are available. Finally, biochronologies benefit from the fact that otolith increments reflect the growth of individual fish over discrete time intervals^13,15^, providing phenotypic measurements that are not available from the traditional size-at-age data. These repeated measurements of phenotypic traits along an environmental gradient give unusual opportunities for separating individual phenotypic plasticity from population-level effects^16,17^.

Phenotypic plasticity is a major mechanism of response to environmental variability, which may allow organisms to cope with rapid shifts, including global climate change^18,19^. Phenotypic plasticity can be expressed as the ability of a single genotype to express a modified phenotype under heterogeneous environmental conditions^6^. Individual phenotypic plasticity is often conceptualized in the form of reaction norms, functions that relate individual phenotypes to an environmental variable^20^. Estimations of individual reaction norms and persistent between‐individual effects due to environmental variability (Fig. 1a–c) are possible with the extension of mixed-effects models applied to biochronological data^21,22^. The slope of the individual’s reaction norm estimated with this statistical approach reflects the magnitude of change in a phenotypic trait across an environmental gradient and is used as a measure of individual phenotypic plasticity^10,23^. Mixed-effects models can also be applied to test for the presence of variation in phenotypic plasticity between individuals^10^ (Fig. 1d–f).

Changes in the variation of individual phenotypic plasticity over time^18^ (Fig. 1g–i) constitute an additional level of biological diversity to be explored in ecological and evolutionary studies^8^. High variation in individual growth plasticity can be considered as one of the elements of “biocomplexity”, which helps to maintain resilience to environmental change^24,25^. The variation of individual phenotypic plasticity may be affected by the environmental conditions^26^ or in the case of commercially exploited fish stocks by fishing pressure^22^. Systematic changes in the variation of individual plasticity are rarely documented in wild populations, even though it has important implications for our understanding of the environmental dependencies of growth under varying conditions^8^.

**Figure 2:**
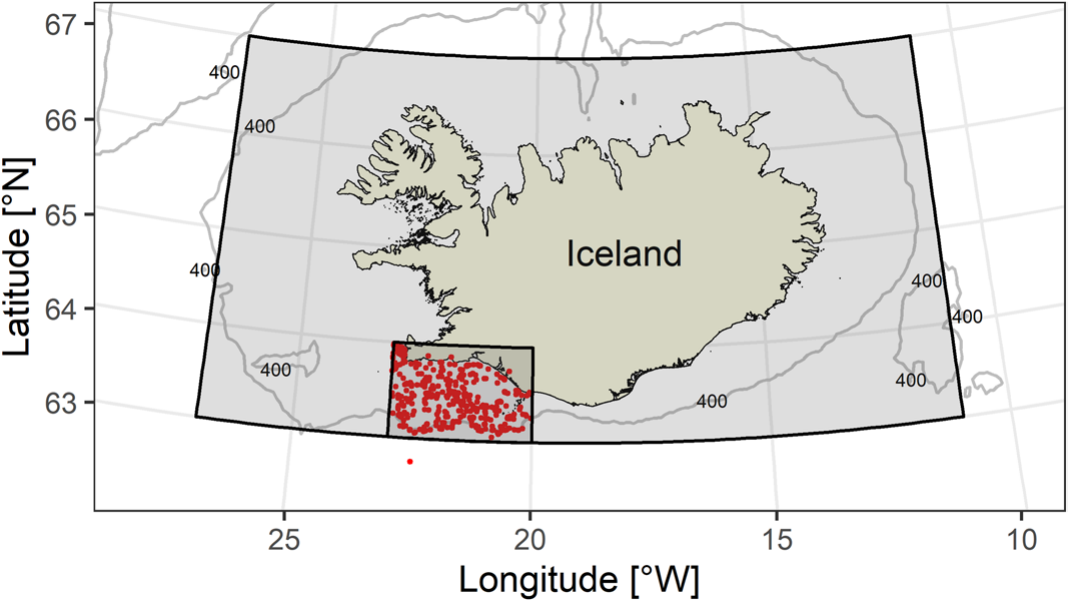
Atlantic cod (*Gadus morhua*) sampling locations (red dots). Three land-based locations reflect errors in the capture location data. Shaded polygons indicate areas (smaller—spawning area and larger—Icelandic shelf) over which sea surface temperature (SST) data were averaged. Isobaths of 400 m are indicated with solid lines. This map was generated using *R* version 3.5.1^65^ based on the GSHHG shoreline database^67^ and the ETOPO1 bathymetric database^68^.

In this study, our objective was to assess individual thermal plasticity of growth in commercially exploited fish species. We hypothesized that population-level responses are driven both by within-individual effects (individual phenotypic plasticity) and among-individual effects (Fig. 1a–c; Hypothesis 1). We also hypothesized that individuals can differ in their thermal plasticity of growth (Fig. 1d–f; Hypothesis 2) and that variation of this individual-level growth plasticity can change between cohorts under natural environmental alterations and human-induced fishing pressures (Fig. 1g–i; Hypothesis 3). To test these hypotheses, we collected measurements of otolith increments from a large historical archive of Atlantic cod (*Gadus morhua*) otoliths from Icelandic waters (Fig. 2). We selected cod because of its key importance in many marine ecosystems^27^ and wide distribution over the whole North Atlantic^28^. The repeated measurements of growth in different environmental conditions for each year of individuals’ life allowed us to investigate individual growth plasticity. We applied linear mixed-effects models on 28,234 otolith increment measurements and developed a century-long biochronology (1908–2014). We controlled for the influence of different intrinsic and extrinsic factors affecting growth to accurately estimate individual fish growth-temperature relationships. We quantified interannual variation and cohort-specific changes in the mean growth of the population and showed that temperature was the main environmental driver of growth. The unique biochronological information on individual fish growth histories over a long time period enabled us to explore individual thermal plasticity of growth and its between‐individual variation.

## Results

### Growth measurements

**Figure 3:**
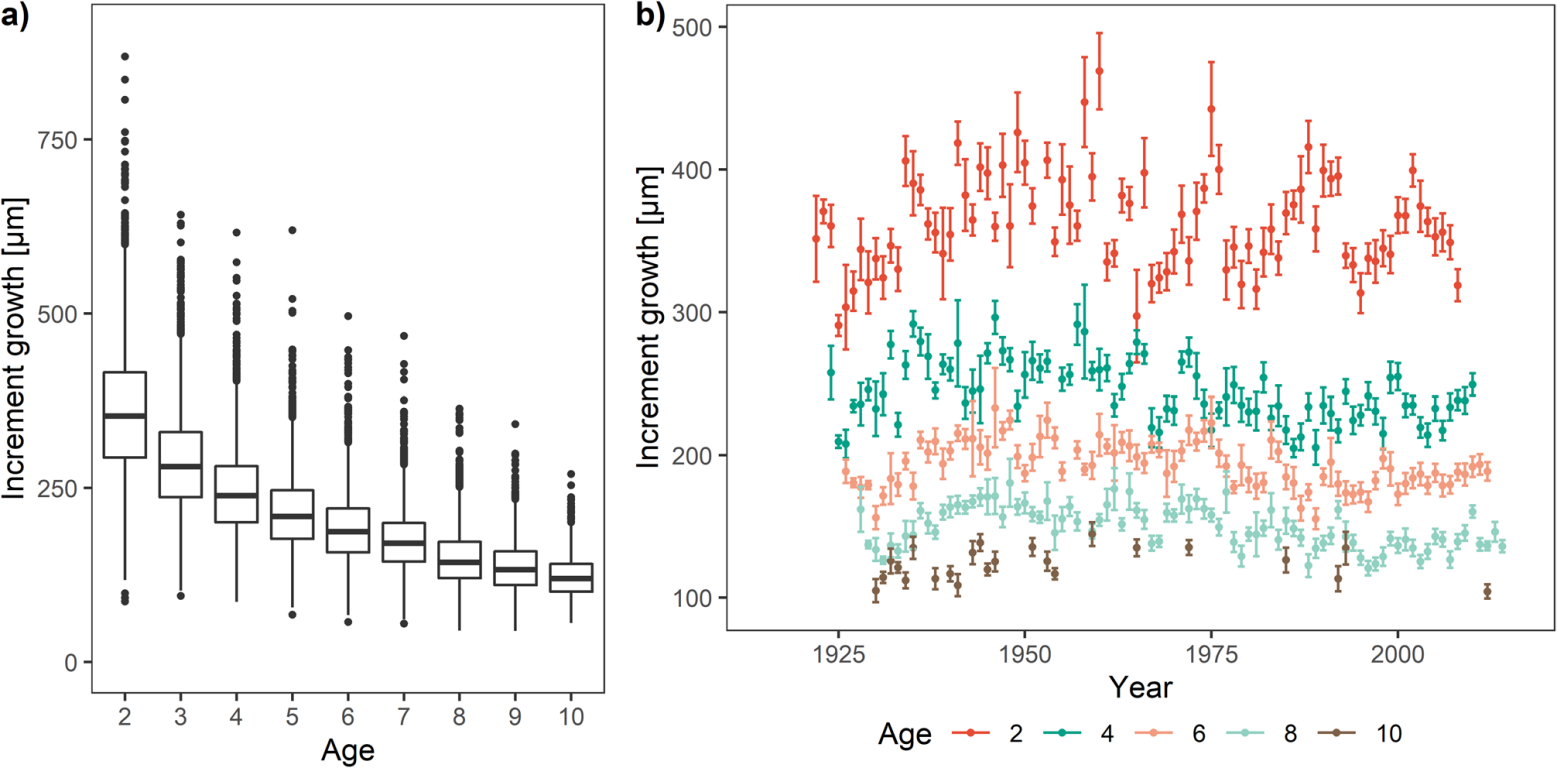
Measured otolith increment width by age classes (**a**; lines, boxes, and whiskers are medians, interquartile range (IQR), and 1.5 IQR, respectively) and time series of mean (± standard error) otolith increment width for the selected age classes (**b**; values are presented only for instances with at least ten observations).

Measured otolith increment widths ranged between 45 and 869 μm with a significant age-dependent negative effect (decrease in width as fish get older, Fig. 3a). There was also an interannual variation in the mean growth of fish within different age classes. We observed wider otolith increments during the period from 1940 to 1970 (Fig. 3b) for all the age classes.

### Intrinsic and extrinsic sources of growth variation

Growth varied significantly between individuals, years and cohorts (Table 1, Supplementary Table S1). A relatively high proportion of variance was associated with the random effect of FishID. The intraclass correlation coefficient of the FishID random intercept-only model was 13.4%. Both Year and Cohort random effects represented a similar level of variance in the model. The intraclass correlation coefficients calculated from intercept-only models were 4.1% for Year random effect and 3.6% for Cohort random effect. The inclusion of Sex effect did not improve the intrinsic model fit (Supplementary Table S2).

**Table 1:** Parameter estimates of the optimal intrinsic, extrinsic, and extended extrinsic model for Atlantic cod growth selected with AICc.

Predictors	Intrinsic		Extrinsic		Extrinsic extended	
	Estimates	CI	Estimates	CI	Estimates	CI
**(a) Fixed effects**						
Intercept	5.326	5.313 to 5.338	5.320	5.307 to 5.333	4.830	4.583 to 5.077
Age	− 0.632	− 0.649 to − 0.615	− 0.646	− 0.666 to − 0.626	− 0.645	− 0.663 to − 0.627
N			0.000	− 0.008 to 0.008	− 0.002	− 0.010 to 0.006
Age:N			0.019	0.005 to 0.033	0.017	0.003 to 0.031
SST_spawn_			0.033	0.009 to 0.058		
Age:SST_spawn_			− 0.068	− 0.098 to − 0.038		
SST_spawn-within_					0.020	− 0.009 to 0.049
Age:SST_spawn-within_					− 0.092	− 0.126 to − 0.058
SST_spawn-among_					0.069	0.035 to 0.103
**(b) Random effects**						
σ^2^	0.056		0.056		0.056	
τ_00_	0.007 _FishID_		0.007 _FishID_		0.007 _FishID_	
	0.002 _Year_		0.002 _Year_		0.002 _Year_	
	0.002 _Cohort_		0.001 _Cohort_		0.001 _Cohort_	
τ_11_	0.013 _Age|FishID_		0.012 _Age|FishID_		0.012 _Age|FishID_	
					0.005 _SSTspawn-within|FishID_	
	0.002 _Age|Year_		0.001 _Age|Year_		0.001 _Age|Year_	
	0.003 _Age|Cohort_		0.006 _Age|Cohort_		0.004 _Age|Cohort_	
ρ	0.399 _FishID-Age_		0.436 _FishID-Age_		0.440 _FishID-Age_	
	− 0.167 _Year-Age_		− 0.050 _Year-Age_		− 0.107 _Year-Age_	
	0.242 _Cohort-Age_		0.286 _Cohort-Age_		0.167 _Cohort-Age_	
N	3728 _FishID_		3677 _FishID_		3677 _FishID_	
	107 _Year_		87 _Year_		87 _Year_	
	100 _Cohort_		89 _Cohort_		89 _Cohort_	
Observations	28,234		26,436		26,436	

Estimates are given for all fixed effects with confidence intervals (*CI*). For the random effects residual variance (*σ*^2^), the variance associated with tested effects (*τ*) and their correlations (*ρ*) are given. The number of observations used to fit the model is specified in the bottom row.

**Figure 4:**
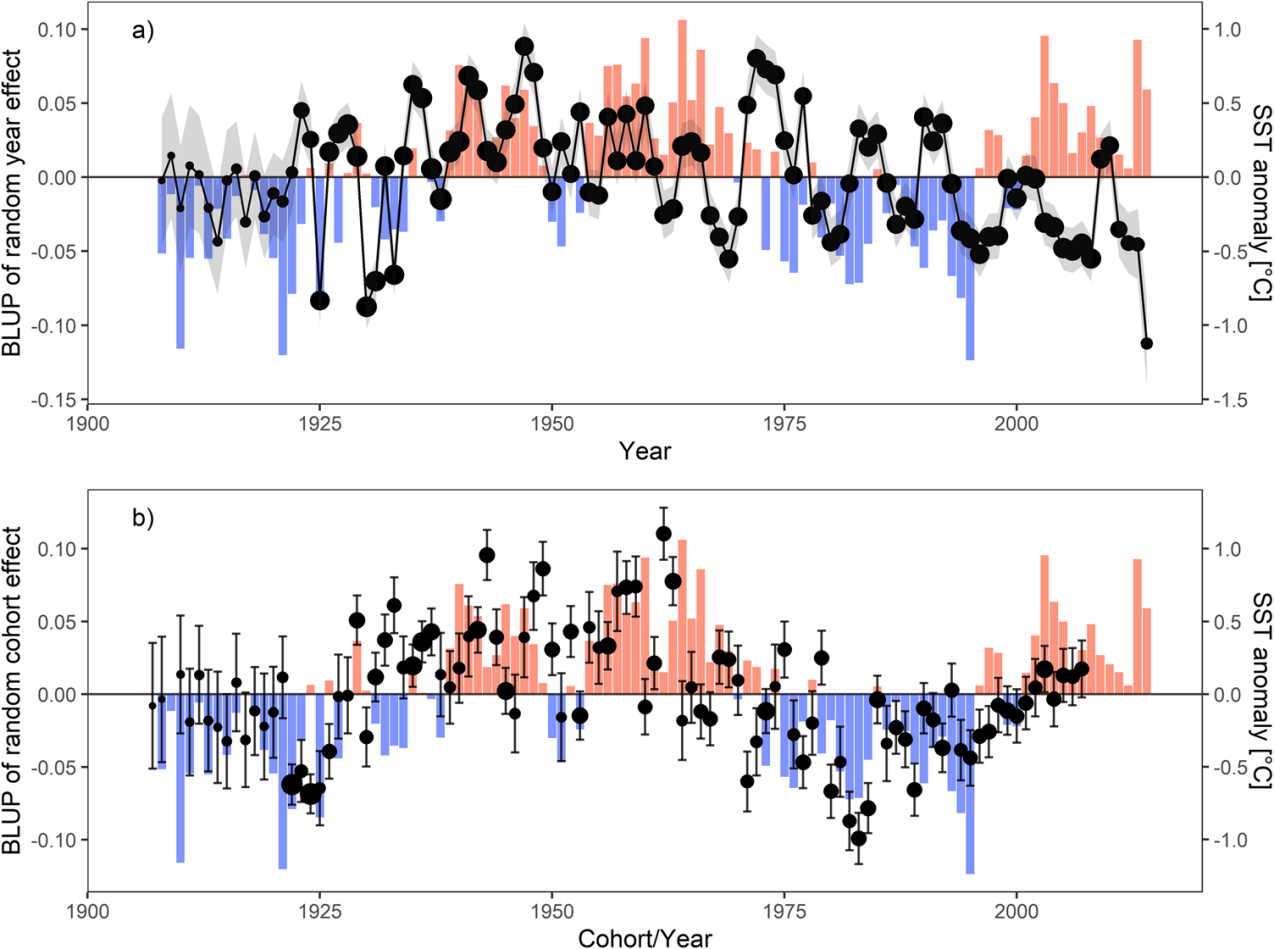
Best linear unbiased predictor (BLUP) of the year (**a**) and cohort (**b**) random effect on Atlantic cod growth and the anomaly of mean April sea surface temperature (SST) within the spawning area. The number of increments measured for each year or cohort is indicated with the size of the dots and standard error of BLUP with the shaded area or bars. Temperature anomaly is indicated with colored bars.

The biochronology record showed considerable year-to-year variation and long-term growth trends (Fig. 4a). Growth was highest in the years 1947 and 1972. Years with especially poor growth were 1925, 1930, and 2014. Cohort-specific patterns of growth were also evident (Fig. 4b). The strongest growth was observed for the individuals from the 1943, 1949 and 1962 cohorts, while fish hatched in the years 1982–1984 were characterized by the lowest growth.

The thermal conditions during the spawning season significantly influenced mean fish growth, the extent of which varied with age and cohort. The systematic sliding-window analysis for temperature variables indicated that the mean April SST in the spawning area (SST_spawn_) in the interaction with Age was the best thermal predictor of cod growth (Supplementary Table S3; Fig. S1). SST_spawn_ was a better predictor than SST_shelf_. Shifts in the SST_spawn_ anomalies were synchronized with changes in both interannual and cohort-specific effects (Fig. 4).

**Figure 5:**
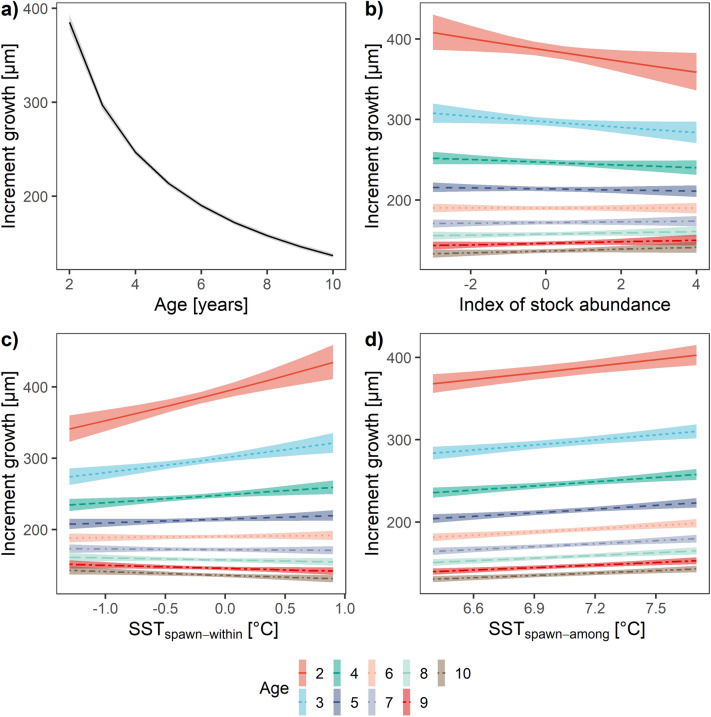
Predicted effect of age (**a**); stock abundance (**b**); within-individual SST differences (**c**); among-individual SST differences (**d**) on cod growth. Shaded areas depict 95% confidence intervals.

Cod growth was also correlated with other environmental conditions, particularly stock abundance (N), in addition to SST_spawn_ (Supplementary Table S4). The extrinsic model of cod growth (Table 1) incorporates SST_spawn_ signal in the interaction with Age and N in interaction with Age, in addition to the intrinsic effect of Age (Fig. 5a). There was no significant effect of harvest rate (HR). The extrinsic model explained about two-thirds of growth variance (marginal R^2^ = 0.57 and conditional R^2^ = 0.66). Comparison of model estimates with the scaled and centered explanatory variables indicated that among the environmental variables SST_spawn_ was the strongest predictor (Supplementary Table S5). Model predictions showed contrasting relationships of SST_spawn_ and growth, depending on age. Fish growth in the 2nd–8th year of life was positively affected by temperature and in the 9th–10th year of life negatively affected by the temperature (Supplementary Table S6). Conversely, growth in the 2nd–5th year of life was negatively affected by N and growth in the 6th–10th year of life was positively affected by N (Fig. 5b, Supplementary Table S6).

#### **Hypothesis 1**

Thermal effects within and among-individuals.

We decomposed the effects of SST_spawn_ on growth observed at the population level into within-individual effects, SST_spawn-within_, and among-individual effects SST_spawn-among_ (Fig. 5c, d). SST_spawn-within_ expresses the plastic responses of the individuals to interannual deviations of the environment from each individual’s lifetime average. SST_spawn-among_ expresses the responses associated with the differences among individuals in the individual’s lifetime average. The level of variance explained by the SST_spawn-within_ represented approximately half of the variance associated with the SST_spawn-among_, reflecting the importance of both effects. Model comparisons with AICc supported the inclusion of SST_spawn-within_ in interaction with Age and SST_spawn-among_ (Supplementary Table S7). Similar to the effects of the original SST_spawn_ variable in the baseline extrinsic model, positive relationships between growth and SST_spawn-within_ were observed for the fish in the 2nd–6th year of life and negative relationships in the 7th–10th year of life (Fig. 5c). There was no support for the inclusion of the interaction of SST_spawn-among_ with Age term and so the model predicted a consistently positive effect across age groups (Fig. 5d; Table 2).

**Table 2:** Effect of the selected environmental variables expressed as % change in growth.

Predictor (range)	Age								
	2	3	4	5	6	7	8	9	10
**April SST**_**spawn-within**_									
(− 1.29 to 0.90 °C)	27.22	17.27	10.69	5.84	2.03	− 1.08	− 3.70	− 5.95	− 7.92
**April SST**_**spawn-among**_									
(6.40 to 7.71 °C)	9.46	9.46	9.46	9.46	9.46	9.46	9.46	9.46	9.46
**N**									
(− 2.55 to 3.54)	− 10.57	− 6.85	− 4.11	− 1.94	− 0.13	1.43	2.80	4.03	5.14

Effects are predicted for discrete age groups by optimal extended extrinsic model within the range of environmental conditions experienced by the Icelandic cod in the years 1928–2014.

#### **Hypothesis 2**

Variation in individual thermal reaction norms.

We recognized a significant variation in individual thermal reaction norms of cod (Table 1). Comparisons of the models with AICc indicated that inclusion of random slopes for the SST_spawn-within_ significantly improved the model fit (Supplementary Table S8). Fish differ individually in the slope of their growth-SST_spawn-within_ relationships (Supplementary Fig. S2). At age 2, the model predicts a positive slope of the growth-SST_spawn-within_ reaction norm for all fish. Individual thermal response in growth at age 2 within the observed range of SST_spawn-within_ (from − 1.29 to 0.90 °C) predicted by the extended extrinsic model varied from 11.40 to 47.60%, while the average change predicted for the whole population was 27.22%. Predicted individual thermal response in growth at Age 10 varied from − 19.37 to 6.83%, while average change predicted for the whole population was − 7.92%.

#### **Hypothesis 3**

Heterogeneity in the variance of the individual plasticity.

**Figure 6:**
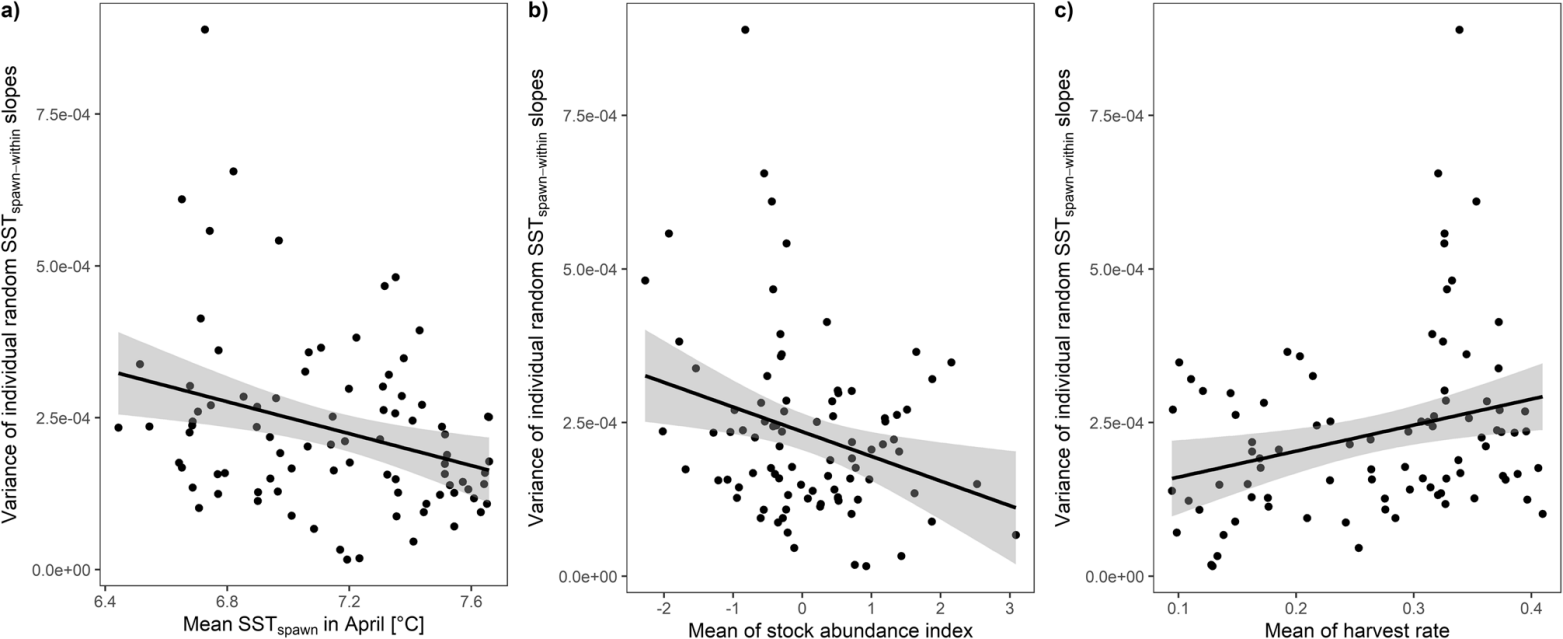
The cohort-specific variance of individual random SST_spawn-within_ slopes regressed with mean SST_spawn_ (**a**), mean stock abundance index (**b**) and mean harvest rate (**c**) experienced by the cohort. Fitted lines are presented with 95% confidence intervals (shaded areas).

We also observed heterogeneity among the cohorts in the variance of the individual plasticity (variance of random SST_spawn-within_ slopes for FishID). Certain cohorts were characterized by more regular growth responses among individuals and homogenized individual plasticity than other cohorts. There was a negative correlation between cohort-specific variance of individual thermal plasticity and the mean SST_spawn_ (ρ =  − 0.31, df = 86, *P* = 0.004), and mean N (ρ =  − 0.28, df = 86, *P* = 0.007) experienced by the cohort (Fig. 6). The variance of individual thermal plasticity was positively correlated with the mean HR (ρ = 0.28, df = 86, *P* = 0.009).

## Discussion

Fish growth is a biological response that integrates many elements – both intrinsic (e.g. ontogeny and sex) and extrinsic (e.g. abiotic conditions of the environment or intra-specific interactions), which may complicate inference about climatic impacts^9^. After accounting for dominating intrinsic age effects, we demonstrated how Icelandic cod growth has varied over the last century. Our main goal was to assess individual plasticity of growth in response to temperature variability, but overlooking non-climate effects in the modeling of fish growth may result in the mistaken assignment of growth changes to particular sources of variation and overestimation of temperature effects^29^. Therefore, we controlled for potential confounding effects of fishing pressure and density-dependence^30^. We selected these potential environmental predictors of the cod growth based on prior ecological knowledge^28,31–34^, taking into consideration data reliability and availability. Some of the potentially important predictors were excluded because the time series of relevant proxies relating to oceanography or prey dynamics are short in comparison with our century-long analysis. For example, the incorporation of time series on abundance of capelin^31^, as well as euphausiids or shrimps, constituting important food resources for Icelandic cod^35,36^, could provide a more complete picture of the environmental drivers of cod growth. The inclusion of large-scale climatic factors (e.g. indices of Atlantic Multidecadal Oscillation or North Atlantic Oscillation) in the analysis could also provide interesting insights on the response of cod growth to lower frequency changes in the environmental conditions^37^, but the incorporation of these indices in the modeling makes it more difficult to accurately estimate more direct effects of temperature on fish growth and thus to investigate individual thermal plasticity.

We found no significant effect of fishing pressure, but a negative influence of the high stock abundance for the growth of fish during their youngest ages and, in contrast, a positive influence during their oldest ages. Density-dependent effects on fish growth are recognized in many populations^38^ and are often studied in Atlantic cod^32^. Higher abundance often leads to enhanced intra-specific competition for limited resources (especially food), causing a decrease in growth^30^. However, density-dependence and competition can impact young and old fish differently^39^. Young cod feed on a limited spectrum of available prey^36^, occupy more constrained space in relatively shallow waters^40^ and their feeding habitat is limited vertically^41^. Moreover, the density of young cod is higher and fluctuates more markedly than in older cod^42^. These biological properties may enhance the intra-specific competition and negative density-dependent effects during the youngest ages. Our model predictions suggested that the effect of stock abundance was positive for fish during their oldest ages, in contrast to previous findings which showed slower growth at high abundances also for the older cod individuals^43^. Positive relationships between stock size and growth can be observed when stock is reduced to the level at which group dynamics and cooperative interactions are weakened, limiting spawning success, foraging, or avoidance of predators^44,45^.

The most important extrinsic predictor of cod growth in our model was the mean SST in the spawning area. Because growth is the integration of a series of processes, such as feeding, assimilation, metabolism, transformation, and excretion, and their rates are all controlled by temperature, this environmental variable was previously considered as the significant controlling factor^46^*.* We identified April as a critical time window of temperature for the prediction of Icelandic cod growth, which coincides with the spawning peak observed from March to May^47^. We observed strong growth of cohorts hatched in the years characterized by warm April conditions, which suggested potential carryover effects associated with the thermal influences during the early life of fish^48^. April SST in southwest Iceland may serve as a predictor of the biological productivity of the weakly stratified Atlantic waters^49,50^. High biological production in spring can cause indirect and lagged (through food supply) effects on cod during the intensive feeding periods^36^. Preliminary analysis revealed the highest correlations between monthly SST and temperature at depth in April. Therefore, April SST provides also a relatively good representation of the interannual variability of thermal conditions at depth, which more directly affects the growth of cod. Our model predictions revealed the strongest and positive temperature effects for the growth during the youngest ages, which is in line with previous findings^33,51,52^. Such age-dependent temperature effects are also in agreement with the general temperature–size rule, where temperature increase leads to faster juvenile growth of fish and smaller adult body sizes^2^.

Our results showed that cod growth variation was associated with the persistent effects of cohorts, which can reflect differences in the systematic response to environmental variability^9^*.* For example, individuals from a particular year class may experience good (or poor) environmental conditions during the juvenile phase and those positive (or negative) effects may be carried over during the life of fish and be manifested in future growth^48^. Such cohort-specific variation in growth was previously found in Icelandic cod^31^, suggesting persistent influences of extrinsic conditions, such as food availability modulated by intraspecific competition. Here, the importance of temperature conditions for the cohort mean growth was supported by the results of the within-group centering of the SST variable. Besides the clear within-individual age-dependent responses, permanent among-individual effects of water temperature were observed (Hypothesis 1). Thus, on average, fish that experienced warmer temperatures during their life grew more. Directional variation in growth was especially visible throughout the chronology in the synchrony of positive and negative phases of cod growth and SST anomalies. These patterns were prominent in both the interannual and cohort-specific changes. In general, there was a phase of significantly higher growth of fish in the warm years approximately 1930–1970s, followed by the colder period 1970s-present when cod growth was lower than average. The last decline in growth was probably caused by a combination of the deteriorating environmental conditions in Icelandic waters, including alterations of the temperature regime and a high stock abundance^43^.

We detected significant variation in individual plasticity of the response to temperature, as seen in the variation in the slope of the thermal reaction norms between individuals (Hypothesis 2). We consider this variation to be meaningful biological information rather than ‘noise’^23,53^. For example, alternative foraging patterns (deep- or shallow-water migrations) in Icelandic cod^47^ may contribute to the observed variation of individual thermal plasticity of growth. Variation in the plastic responses of the individuals to climatic variability has implications for the way we interpret trait–environment relationships in organisms^8^. Such individual differences in phenotypic plasticity can arise from genetic effects^54,55^, but also from early-life or current environmental conditions^19^. When a heritable (genetic) component is present in the variation of the individual phenotypic plasticity, potentially it can evolve under directional selection^26^. Quantifying the genetic basis of reaction norms properties is possible with the application of the mixed effect modelling framework extended to the so-called ‘animal model’ incorporating relatedness estimates^10,23^. Additional studies involving the suitable measure of individual fish fitness (e.g. reproductive success) in order to assess the consequences of variation of plasticity and its adaptive, non-adaptive, or maladaptive nature would be valuable^6,10^. There is still relatively scarce knowledge about how selection acts on plasticity in wild animal populations^53,56^. Since potential evolutionary changes in the growth plasticity of fish can lead to alterations in the reaction norms under new environmental conditions, identification of such changes is of high importance for our predictions of the population responses to the changing global climate^18^.

We highlighted that the variance of thermal plasticity was heterogeneous across fish from different cohorts (Hypothesis 3). Cohorts that experienced—on average—warmer conditions had less variable individual growth plasticity to the temperature changes than cohorts living in the colder periods. Within the range observed in this study, higher temperatures (10–15 °C) are closer to the optimum of the species^28,51,52^. Therefore, one may expect that lower temperatures will result in more regular growth responses and homogenized individual plasticity due to the more pronounced thermal stress, but this was not the case. We observed also that variance of individual growth plasticity was lower at the higher stock abundance. The increase in abundance of stock corresponds to the decline in environmental quality due to the increased competition^30,32^. This in turn, likely reduces the possibilities of exploiting different strategies for the allocation of surplus energy between reproduction or somatic growth^57^ and results in the homogenization of the individual plastic responses. Similar mechanisms may explain the positive relationship between harvest rate and cohort-specific variance of individual phenotypic plasticity. High harvest rate, through the release from density dependence^30^ and improvement of fish physiological conditions, may create capacity for the variation in individual phenotypic plasticity. However, harvesting can also diminish the expression of phenotypic diversity within populations as a direct effect of selection or the disruption of social hierarchies^22^.

The between-individual variation in phenotypic plasticity underlying observed trait–environment relationships are rarely considered due to the large, long-term datasets required for such analysis^8,16^. Observational data collected for the wild populations only allow for a correlative approach, and other unmeasured variables may play a role in shaping variation in plastic responses^18^. Therefore, the underlying ecological and physiological mechanisms of the heterogeneity in the variance of the individual plasticity observed among cod cohorts remain unclear. Nevertheless, we present unique results from a natural, managed population indicating that individual responses to temperature changes are notably variable, and it is likely that the variance of individual growth plasticity may change among cohorts under different environmental conditions. Some cohorts may be constrained by the environmental conditions in expressing phenotypic plasticity^26,56^. Thus, our ability to detect variance in the phenotypic plasticity at the individual level may depend on the environmental conditions experienced by the studied cohort^8^. This evidence of between-individual variation in the thermal reaction norms is an important contribution to our understanding the complexity of plastic responses among organisms and the consequences for coping with current and future climate change^11,26^.

## Materials and methods

### Otolith sampling and measurements

We selected cod otoliths from the archival collection of the Marine and Freshwater Research Institute (MFRI) in Iceland (no fish were caught for the purpose of the experiment). After a preliminary analysis of gear selectivity on fish length at age, we included only fish caught with longlines and bottom trawls. We selected otoliths from fish taken in commercial catches and scientific surveys sampled in the years 1929–2015 in the marine areas of southwest Iceland (Fig. 2; Supplementary Fig. S3). In order to capture growth patterns of multiple cohorts^15^ we randomly selected 50 otoliths of fish age 8 (the most abundant of the mature age groups) or older and 3 individuals of age 10 or older from each year. In total, we included 3728 otoliths for growth analyses (Supplementary Fig. S4).

The otoliths were embedded in black epoxy and sectioned to a thickness of 1 mm with a Buehler IsoMet 1000 Precision Saw with a diamond blade. We photographed the sections using a high-resolution digital camera Olympus DP74 connected to a stereomicroscope Leica S8 APO (apochromatic 8:1 zoom, high magnification up to 80x) under reflected light. CellSens Standard v. 1.18 and CellSens Dimension v. 1.18 software (https://www.olympus-lifescience.com/en/software/cellsens) was used for the image capture and editing, and for increment measurements.

The selection of the otoliths was based on the historical age estimates recorded in the MFRI database, which involved several age readers over the past century. As part of the biochronology measurements, all otoliths were subsequently re-aged by a single age reader (JDL) from otolith section images. Finally, to better assess the internal consistency of the age estimates, an expert cod otolith age reader re-aged ~ 24% of a random sample of the otolith images across the entire time series (N = 906). Re-ageing by a single experienced age reader allowed for the detection of possible confounding effects due to changes in ageing method or age reader over time. This re-ageing confirmed the absence of appreciable ageing bias between original and new age estimates through time, as well as a reasonably high ageing precision (coefficient of variation = 3.2%).

We measured growth increment widths along the distal axis marking the measurements on the medial edge of each translucent zone in order to ensure recognition of the last increment before the edge (Supplementary Fig. S5). Because the position of the core was not clear for every section, we marked the longest diameter of the first increment, then drew a line perpendicular to the growth increments along the distal axis and used the crossing point between this line and the diameter as the origin for the measurements. The total length of cod was strongly correlated with otolith width (Supplementary Fig. S6). Therefore, we assumed that otolith growth is proportional to somatic growth and applied otolith increment data as a proxy to directly reconstruct annual growth histories of individual fish. We excluded from the analysis the small number of measurements of the increments formed after the 10th year of the fish life. We also excluded measurements of the first and last (edge) increments because they did not represent complete growth through the year. In total, our analyses used 28,234 measurements of the otolith increments formed in the years 1908–2014 (Supplementary Fig. S7).

### Predictors of fish growth

We selected potential intrinsic and extrinsic predictors of cod growth variation (Table 3). We considered systematic differences between fish individuals (FishID), years of otolith increment formation (Year) or groups of individual fish hatched in the same year (Cohort), treated as random effects. Fixed intrinsic variables included Age and Sex. We used mean monthly Sea Surface Temperature (SST) from HadISST data^58^ within the main spawning area (63°N to 64°N, − 23°E to − 20°E) and whole Icelandic shelf (63°N to 67°N, − 27°E to − 11°E), as representing stock area^59^ (Fig. 2; Supplementary Fig. S8). Although bottom water temperature would have been a preferable index of temperature exposure for the cod, the SST time series was used since it covered the entire time span of the otolith biochronology, and gridded HadISST data allowed for the additional analysis of spatial correlations. Annual mean SST and mean ocean temperature (0–700 m layers, World Ocean Database of the National Oceanic and Atmospheric Administration) in the years 1956–2014 in the Icelandic shelf area were reasonably well correlated (R = 0.83, df = 57, *p* < 0.001), therefore, SST was considered a sufficient proxy for the thermal conditions experienced by the cod. Additional preliminary analysis on the limited data set showed that SST was a stronger predictor of cod growth than mean ocean temperature.

**Table 3:** List of predictors of Atlantic cod growth.

Predictor	Description
**Random effects**	
FishID	Unique identifier of the fish individual
Year	Calendar year of otolith increment formation
Cohort	Group of fish from the same spawning season
**Fixed effects**	
Age	Age of fish when growth increment was formed
Sex	Sex of the individual
SST_spawn_, SST_shelf_	Mean monthly sea surface temperature data^58^ (1901–2014), aggregated over the main spawning area (subscript spawn) or Icelandic shelf (subscript shelf)
N	Yearly data (1928–2014) on cod stock abundance of the age group, the results of the extended virtual population analysis, see Supplementary Note 1
HR	Yearly data (1928–2014) on cod stock harvest rate (proportion of the fish harvested from the stock), the results of the extended virtual population analysis, see Supplementary Note 1

Overlooking other extrinsic non-climatic effects in the modeling of fish growth may result in the mistaken assignment of growth changes to particular sources of variation and overestimation of climatic effects^29^. Thus, we included the annual abundance of fish at age groups (N) from the extended virtual population analysis (see Supplementary Note 1) as an index of stock abundance to control for density-dependent effects. We calculated the index by scaling the numbers within the age groups after logarithmic transformation (Supplementary Fig. S8), providing unique values for the given year and given age^34^. We used harvest rate (HR), estimated in the extended virtual population analysis as the rate of observed yield to the biomass of 4 years and older fish to control the effects of harvesting, or fishing pressure, on growth.

### Data analysis

Prior to the modeling, we log-transformed Increment width and Age to satisfy model assumptions, and mean-centered all continuous explanatory variables. We developed a series of mixed-effects models to test potential intrinsic and extrinsic sources of variation in fish growth^9^. This modeling approach takes into account the hierarchical structure of the biochronological data (repeated measurements of otolith annual increments from one individual, year or cohort) and allows for robust assessment of different sources of variation^60^.

In the first step, we determined the optimal random effect structure by comparison of models including different combinations of random intercepts for FishID, Year or Cohort, and random Age slopes for these three terms. The following formula was used to fit the intrinsic and extrinsic model:

$$\begin{aligned} y_{ijkl} & = \alpha_{0} + \alpha_{i}^{F} + \alpha_{k}^{Y} + \alpha_{l}^{C} + \beta_{j} x_{j} + b_{ij}^{F} x_{ij} + b_{jk}^{Y} x_{jk} + b_{jl}^{C} x_{jl} + f\left( \cdot \right) + \varepsilon_{ijkl} \\ \left[ {\begin{array}{*{20}c} {\alpha_{i}^{F} } \\ {b_{ji}^{F} } \\ \end{array} } \right] & \sim N \left( {0, \sum_{i} } \right),\quad \left[ {\begin{array}{*{20}c} {\alpha_{k}^{Y} } \\ {b_{jk}^{Y} } \\ \end{array} } \right]\sim N \left( {0, \sum_{k} } \right),\quad \left[ {\begin{array}{*{20}c} {\alpha_{l}^{C} } \\ {b_{jl}^{C} } \\ \end{array} } \right]\sim N \left( {0, \sum_{l} } \right),\quad \varepsilon_{ijkl} \sim N \left( {0,\sigma^{2} } \right) \\ \end{aligned}$$

where *y*_*ijkl*_, annual growth *y* for fish *i* at age *j* (j = 2, …, 10) from year *k *(*k* = 1908, …, 2014) and cohort *l *(*l* = 1907, …, 2007), *α*_0_ is the overall growth intercept, $\alpha_{i}^{F}$ is the random intercept for fish *i*, $\alpha_{k}^{Y}$ is the random intercept for year *k*, $\alpha_{l}^{C}$ is the random intercept for cohort *l*, $\beta_{1} x_{ij}$ is the age-dependent (*j*) decline in growth, $b_{ij}^{F} x_{ij}$ is the random age (*j*) slope for fish *i*, $b_{jk}^{Y} x_{jk}$ is the random age (*j*) slope for year *k* , $b_{jl}^{C} x_{jl}$ is the random age (*j*) slope for cohort *l*, *f*(·) indicates fixed effects and their interactions with age (*j*). In this stage, models were fitted including all potential intrinsic effects (Age in interaction with Sex). We compared models with the Akaike information criterion corrected for the small sample sizes (AICc). Further, we identified optimal fixed intrinsic factors based on AICc comparisons of models with varying fixed effects complexity, while keeping the previously selected best-ranked random effect structure. We used the best linear unbiased predictors (BLUP) of the random effects to visualize the temporal patterns of fish growth variation. We extracted the BLUP from the best-ranked intrinsic model which did not incorporate random Age slopes for the Year or Cohort effects. By excluding the Age random slopes, we preserved any possible long-term changes of the age-growth relationships in the extracted BLUP time series.

In the second step, we introduced different extrinsic effects into the optimal intrinsic model identified in the first step of modeling. In order to make a non-arbitrary choice of the optimal time window for the monthly temperature variable (SST), we applied statistically-based sliding window analysis, similarly to previous biochronological studies^5,61^. We included mean values of investigated variables calculated from different absolute time windows within the 24 months counted back from the end of the growth year (December) in the subsequent models and compared these to the baseline intrinsic model treated as a null hypothesis^62^. This approach allows one to test possible environmental signals affecting fish growth with different time lags^61^. We included temperature variables both with and without Age interaction and assumed a linear relationship. We identified the optimal predictor (variable with a critical time window) based on AICc results. Further, we ran 999 randomized iterations in which we reordered the date variable paired to the response (increment width) and removed any dependency between extrinsic variables and fish growth^62^. We compared the AICc result from the analysis to the AICc distribution of randomized models to quantify the likelihood of obtaining such model support by chance^63^.

After identification of the optimal time window for SST based on the whole dataset (28,234 measurements for the years 1908–2014), remaining potential growth predictors (N and HR) were tested in the model with and without Age-interaction. Models for this comparison were developed based on a subset of data (26,436 measurements for the years 1928–2014) due to the shorter time span of N and HR time series. The selected optimal extrinsic model was refitted with the scaled and centered response and explanatory variables in order to compare the relative effects of intrinsic and extrinsic environmental factors on fish growth.

In the third step, we used within-group centering^21^ to determine if average population growth response to the temperature variability was dominated by a within-individual or an among-individual effect, i.e. individual phenotypic plasticity or among-individual differences in the environmental conditions^9,44^. Using this technique, we replaced a temperature variable in the model by two new variables: (1) the average temperature conditions experienced by individuals across their lifetime and (2) the deviations of temperature from this mean; and compared to the baseline extrinsic model using AICc. Further, we replaced within-individual deviations with an original temperature variable to investigate differences in within-individual and among-individual effects^21^.

In the fourth step, we extended the optimal extrinsic model by the addition of random slopes of the within-individual component of the temperature variable for FishID^53^. The following formula was used to fit the extended extrinsic model:

$$\begin{aligned} y_{ijkl} & = \alpha_{0} + \alpha_{i}^{F} + \alpha_{k}^{Y} + \alpha_{l}^{C} + \beta_{1} x_{ij} + b_{1i}^{F} x_{ij} + b_{1k}^{Y} x_{jk} + b_{1l}^{C} x_{jl} + \beta_{w} (x_{ik} - \overline{x}_{ik} ) \\ & \quad + b_{wi}^{F} (x_{ik} - \overline{x}_{ik} ) + \beta_{A} \overline{x}_{ik} + f\left( \cdot \right) + \varepsilon_{ijkl} \\ \left[ {\begin{array}{*{20}c} {\alpha_{i}^{F} } \\ {b_{ji}^{F} } \\ \end{array} } \right]\sim & N \left( {0, \sum_{i} } \right),\quad \left[ {\begin{array}{*{20}c} {\alpha_{k}^{Y} } \\ {b_{jk}^{Y} } \\ \end{array} } \right]\sim N \left( {0, \sum_{k} } \right),\quad \left[ {\begin{array}{*{20}c} {\alpha_{l}^{C} } \\ {b_{jl}^{C} } \\ \end{array} } \right]\sim N \left( {0, \sum_{l} } \right),\quad b_{wi}^{F} \sim N \left( {0,\sigma_{F}^{2} } \right),\quad \varepsilon_{ijkl} \sim N \left( {0,\sigma^{2} } \right) \\ \end{aligned}$$

where $\beta_{w} (x_{ik} - \overline{x}_{ik} )$ is the within-individual temperature slope, $\beta_{A} \overline{x}_{ik}$ is the among-individual temperature slope, $b_{wi}^{F} (x_{ik} - \overline{x}_{ik} )$ is the random within-individual slope. With a mean-centered environmental variables in the model, the intercept equates to the expected growth of the individual in the average environment, while the slope parameter estimates the change in growth across an environmental gradient and is, therefore, a measure of individual phenotypic plasticity^10,23^. Using this approach we examined variance and covariance in the random intercepts and linear slopes of individual fish responses to the changes in the environmental conditions^56^. Further, using a simple Pearson correlation we investigated possible relationships between the cohort-specific variance of individuals’ thermal plasticity (BLUP of individual thermal reaction norm slopes) with mean environmental conditions experienced by the cohort^26,64^. Only cohorts represented by more than five individuals were included.

Models during random effects optimization were fitted using a restricted maximal likelihood (REML), while fixed effects optimization used a maximal likelihood approach. The optimal models were then refitted with REML in order to obtain unbiased parameter estimates. We checked and satisfied assumptions of the final model with standard diagnostics and tested multicollinearity of the explanatory variables (variance inflation factors less than 2). We calculated intraclass correlation coefficients of the intrinsic intercept-only models to assess the level of correlation between fish growth within individuals, years or cohorts, while the conditional and marginal R^2^ metric of the final model to assess the variance in fish growth explained by both fixed and random effects^9^. Data on water temperature were extracted from the Royal Netherlands Meteorological Institute Climate Explorer website (https://climexp.knmi.nl). We used the *R* scientific computing language^65^ with *lme4*^66^ and *climwin* packages^62^ for the data analysis.

## Supplementary information

Supplementary information.

## Data Availability

The datasets generated and analyzed during the current study are available from the corresponding author on reasonable request.
