# Supplementary material for: Century-long cod otolith biochronology reveals individual growth plasticity in response to temperature
Source: Sci Rep. 2020 Oct 7;10:16708. doi: 10.1038/s41598-020-73652-6 (PMC7541619; doi:10.1038/s41598-020-73652-6)
Supplement: Supplementary file 1 — Supplementary information. [file 41598_2020_73652_MOESM1_ESM.pdf]

Supplementary materials:

Century-long cod otolith biochronology reveals individual growth plasticity in response to temperature

Szymon Smoliński[1,*], Julie Deplanque-Lasserre[2], Einar Hjörleifsson[3], Audrey J Geffen[1,4], Jane A Godiksen[1], Steven E Campana[2]

[1]Institute of Marine Research, P.O. Box 1870 Nordnes, N-5817 Bergen, Norway

[2]Faculty of Life and Environmental Sciences, University of Iceland, Reykjavik, Iceland

[3]Marine and Freshwater Research Institute, Reykjavík, Iceland

[4]Department of Biological Sciences, University of Bergen (UiB), Norway

*contact information: szymon.smolinski@hi.no, +4794847852

Supplementary results

Table S1. Selection of the optimal random effects of the Atlantic cod growth model. Series of models were fitted to the data with the full intrinsic fixed-effects structure (age in the interaction with sex). Based on Akaike Information Criterion corrected for the small sample sizes (AICc) the best model was selected (in bold). The random Age slopes for each FishID, Year or Cohort are denoted by "|", and "+" indicates that random term was included in the given model.

Random intercept			Random slope					
FishID	Year	Cohort	Age|FishID	Age|Year	Age|Cohort	df	AICc	ΔAICc
+						8	4298.57	1462.34
+			+			10	3957.28	1121.05
+	+		+			11	3125.46	289.23
+		+	+			11	3353.24	517.01
+	+		+	+		13	2983.22	146.99
+		+	+		+	13	3183.24	347.02
+	+	+	+			12	2966.24	130.01
+	+	+	+	+		14	2868.81	32.59
+	+	+	+		+	14	2877.07	40.85
+	+	+	+	+	+	**16**	**2836.23**	**0**

Table S2. Selection of the optimal fixed intrinsic effects of the Atlantic cod growth model. Series of models were fitted to the data with the optimal random effects structure (Table S1). Based on Akaike Information Criterion corrected for the small sample sizes (AICc) the best model was selected (in bold). Parameter estimates of continuous variables are given in the selection table, “Age:Sex” indicates interaction, and “+” indicates that a term was included in the given model.

Intercept	Age	Sex	Age:Sex	df	AICc	ΔAICc
**5.33**	**-0.63**			**12**	**2780.74**	**0**
5.32	-0.63	+		14	2784.47	3.73
5.32	-0.63	+	+	16	2787.37	6.63
5.26				11	3182.62	401.88
5.26		+		13	3186.40	405.66

Table S3. Results of the sliding window analysis for the identification of the optimal time window of sea surface temperature. “-1y” indicates the year prior to the assigned calendar year of the otolith increment formation. The best model is highlighted in bold.

Term	Age interaction	Window Open	Window Close	ΔAICc
SSTshelf		November-1y	May	-11.67
SSTspawn		March	April	-9.99
SSTshelf	+	March	April	-27.91
**SSTspawn**	+	**April**	**April**	**-30.17**

Table S4. Selection of the optimal fixed extrinsic effects of the Atlantic cod growth model. Series of models were fitted to the data with the optimal fixed intrinsic and random effects structure (Tables S1, S2) and optimal time window of sea surface temperature (Table S3). Based on Akaike Information Criterion corrected for the small sample sizes (AICc) the best model was selected (in bold). Parameter estimates are given in the selection table, ":" indicates interaction.

Intercept	Age	SSTspawn	Age:SSTspawn	N	Age:N	HR	Age:HR	df	AICc	ΔAICc
5.32	-0.65	0.03	-0.07	-0.0011	0.02	-0.10		17	2741.23	0
**5.32**	**-0.65**	**0.03**	**-0.07**	**-0.0001**	**0.02**			**16**	**2741.29**	**0.06**
5.32	-0.64	0.03	-0.06			-0.11		15	2742.73	1.50
5.32	-0.65	0.03	-0.07	-0.0013	0.02	-0.10	-0.02	18	2743.17	1.94
5.32	-0.64	0.03	-0.06					14	2743.35	2.12
5.32	-0.64	0.03	-0.07			-0.11	-0.05	16	2744.36	3.13
5.32	-0.64	0.03	-0.06	-0.0014		-0.11		16	2744.61	3.38
5.32	-0.64	0.03	-0.06	-0.0003				15	2745.35	4.12
5.32	-0.64	0.03	-0.07	-0.0021		-0.12	-0.06	17	2746.12	4.89
5.32	-0.64	0.03				-0.10		14	2755.99	14.76
5.32	-0.65	0.03		-0.0027	0.01	-0.10		16	2756.22	14.99
5.32	-0.64	0.04						13	2756.35	15.12
5.32	-0.64	0.04		-0.0018	0.01			15	2756.35	15.12
5.32	-0.64	0.03		-0.0030		-0.11		15	2757.47	16.24
5.32	-0.65	0.03		-0.0019	0.01	-0.09	0.07	17	2757.68	16.45
5.32	-0.64	0.03				-0.10	0.04	15	2757.78	16.56
5.32	-0.64	0.04		-0.0020				14	2758.12	16.89
5.32	-0.65			-0.0027	0.02	-0.14		15	2758.22	16.99
5.32	-0.64					-0.14		13	2759.11	17.88
5.32	-0.64	0.03		-0.0027		-0.11	0.03	16	2759.39	18.16
5.32	-0.65			-0.0018	0.02	-0.13	0.07	16	2759.60	18.37
5.32	-0.64			-0.0013	0.02			14	2760.19	18.96
5.32	-0.64			-0.0030		-0.15		14	2760.61	19.38
5.32	-0.64					-0.14	0.04	14	2760.90	19.67
5.32	-0.64							12	2761.79	20.56
5.32	-0.64			-0.0027		-0.15	0.03	15	2762.53	21.30
5.32	-0.64			-0.0015				13	2763.68	22.46

Table S5. Estimates of fixed effects provided by optimal extrinsic model (Tables S1, S2, S3, S4) with the scaled and centered response and explanatory variables. “CI” - confidence intervals for the fixed effect estimates, “:” indicates interaction.

Term	Estimates	CI
Intercept	0.003	-0.029 – 0.035
Age	-0.753	-0.776 – -0.730
SST_{spawn}	0.038	0.010 – 0.066
Age:SST_{spawn}	-0.037	-0.053 – -0.020
N	-0.001	-0.023 – 0.022
Age:N	0.025	0.006 – 0.044

Table S6. Effect of the selected environmental variables expressed as % change in growth. Effects are predicted for discrete age groups by optimal extrinsic model within the range of environmental conditions experienced by the Icelandic cod in the years 1928-2014.

Predictor (range)	Age								
	2	3	4	5	6	7	8	9	10
April SST_{spawn} (5.82 to 8.12 °C)	25.91	18.19	13.00	9.14	6.08	3.55	1.42	-0.43	-2.05
N – stock abundance index (-2.55 to 3.54)	-10.86	-6.59	-3.43	-0.91	1.19	3.01	4.61	6.04	7.34

Table S7. Comparison of combined, within and between-individual thermal effects on Atlantic cod growth. Series of models were fitted to the data with the optimal fixed and random effects structure and optimal time window of sea surface temperature (Tables S1, S2, S3, S4). New temperature variables were calculated as: i) the average temperature conditions experienced by individuals across their lifetime ($SST_{spawn\text{-}among}$) and ii) the deviations of temperature from this mean ($SST_{spawn\text{-}within}$) and added as fixed predictors (see van de Pol and Wright 2009 for details on the method). The best model was selected (in bold) based on Akaike Information Criterion corrected for the small sample sizes (AICc). Parameter estimates for the temperature variables are given in the selection table, ":" indicates interaction. For clarity only estimates of temperature-related explanatory variables were included in the table. The bottom row model serves as an additional test of within- and between-individual effects difference. Among-individual effects in this model ($SST_{spawn\text{-}among}$) actually represent the difference between the among- and within-individual effects ($SST_{spawn\text{-}among}$ - $SST_{spawn\text{-}within}$). Thus the estimate of $SST_{spawn\text{-}among}$ - $SST_{spawn\text{-}within}$ is expected to be close to zero when the within- and among-individual effects are effectively the same (van de Pol and Wright 2009).

SSTspawn	Age: SSTspawn	SSTspawn-within	Age: SSTspawn-within	SSTspawn-among	Age: SSTspawn-among	df	AICc	ΔAICc
0.03	-0.07					16	2741.28	17.03
		0.01		0.07		16	2751.29	27.04
		**0.02**	**-0.09**	**0.07**		17	2724.26	0
		0.01		0.07	0.05	17	2749.44	25.18
		0.02	-0.09	0.07	0.03	18	2724.82	0.57
0.02	-0.09			0.05	0.12	18	2724.82	0.57

Table S8. Selection of the optimal random effect structure to test for individual differences in growth plasticity. Series of models were fitted to the data with the previously identified optimal fixed and random effects structure (Tables S1, S2, S3, S4, S7) and added random slope that describe the individual reaction norm of growth versus temperature (SST_{spawn}) allowing for different covariance structures of random intercepts and slopes. The best model was selected (in bold) based on Akaike Information Criterion corrected for the small sample sizes (AICc). The first model is the optimal model from Table S7 refitted with restricted maximal likelihood (REML).

Random FishID slopes	Covariances	df	AICc	ΔAICc
Age	Age (slope) - FishID (intercept)	17	2776.64	4.26
Age, $SST_{spawn\text{-}within}$	Age (slope) - FishID (intercept); Age (slope) - $SST_{spawn\text{-}within}$ (slope); $SST_{spawn\text{-}within}$ (slope) - FishID (intercept)	20	2774.77	2.40
Age, $SST_{spawn\text{-}within}$	Age (slope) - FishID (intercept); $SST_{spawn\text{-}within}$ (slope) - FishID (intercept)	20	2774.85	2.47
**Age, $SST_{spawn\text{-}within}$**	**Age (slope) - FishID (intercept)**	**18**	**2772.38**	**0**

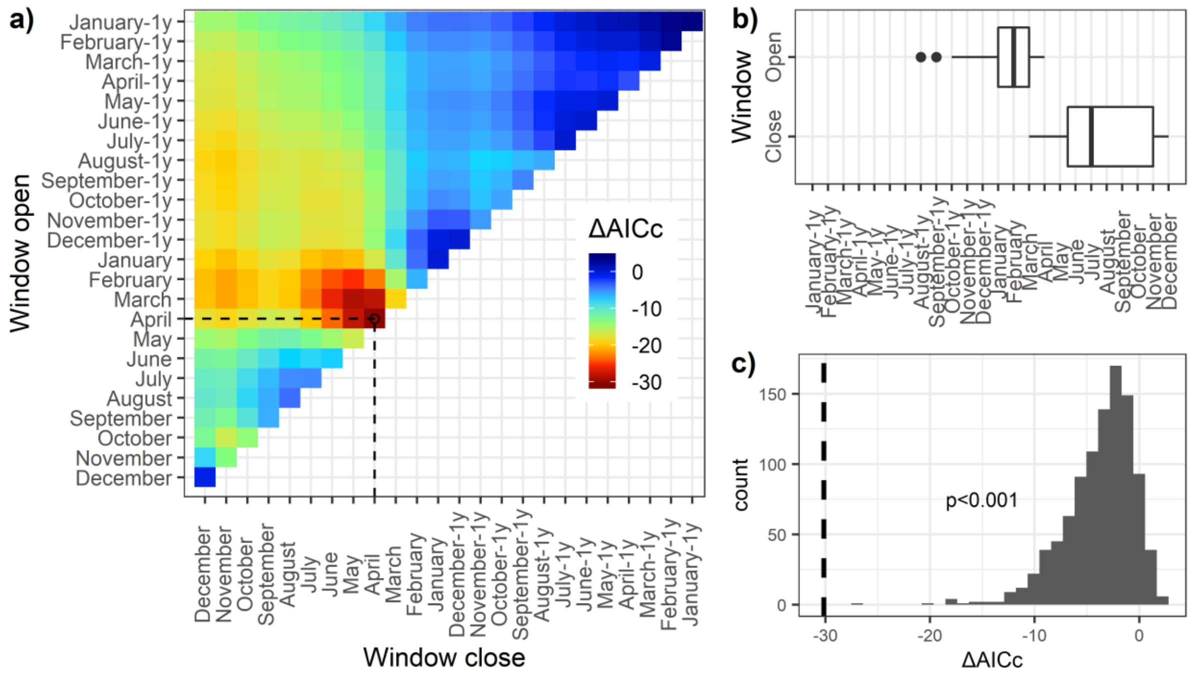

Fig. S1. Results of optimal time window identification for the sea surface temperature (SST) in the spawning area in the year of growth. The months of the window opening or closing are indicated on the axes. ΔAICc (shown with the gradient) is the differences in AICc between the baseline intrinsic model and model with added SST term (a); the opening and closing points of the time windows within the 95% confidence set based on the AIC model weights (b); histogram of the randomized AIC distribution and the final AIC result obtained during the sliding-window analysis indicated with vertical dashed line (c).

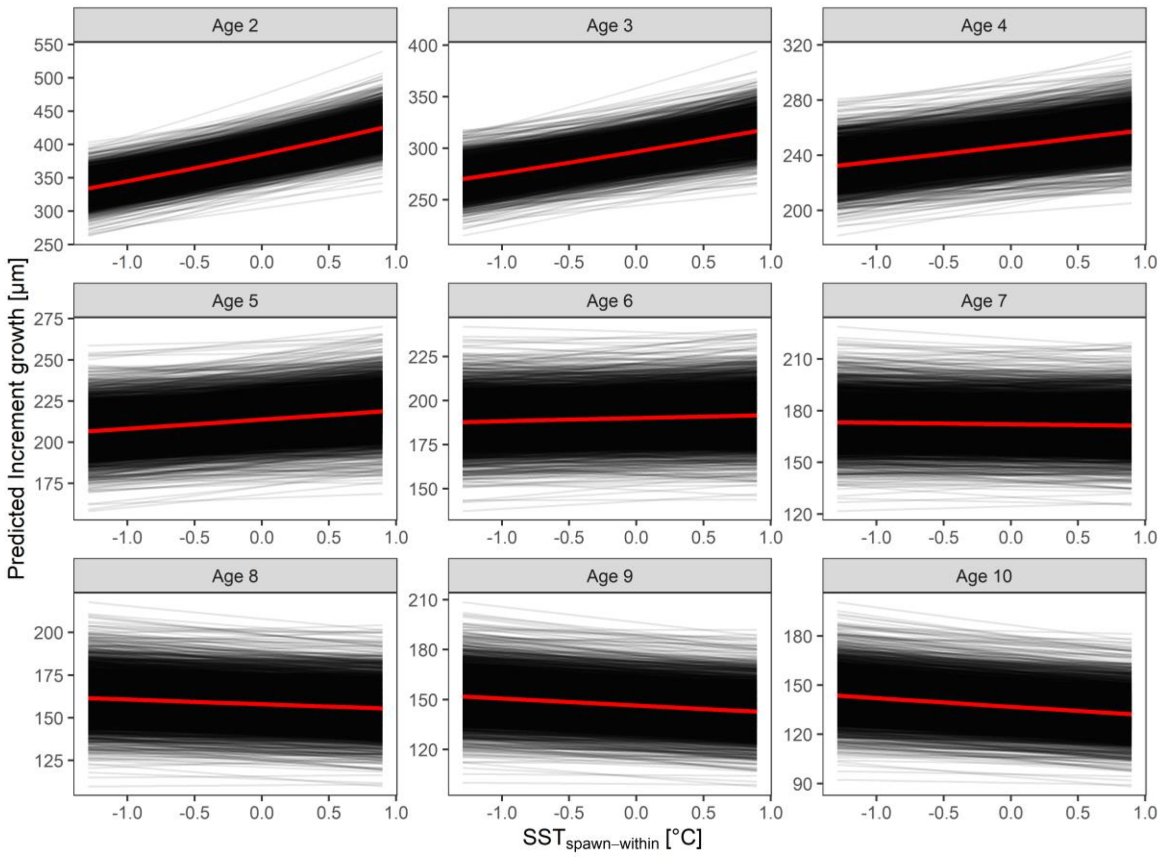

Fig. S2. Individual thermal reaction norms of Icelandic cod. Each panel depicts reaction norms predicted specifically for the analyzed fish ages (from 2 to 10). Notice different scales on the y-axis.

Otolith sampling and measurements

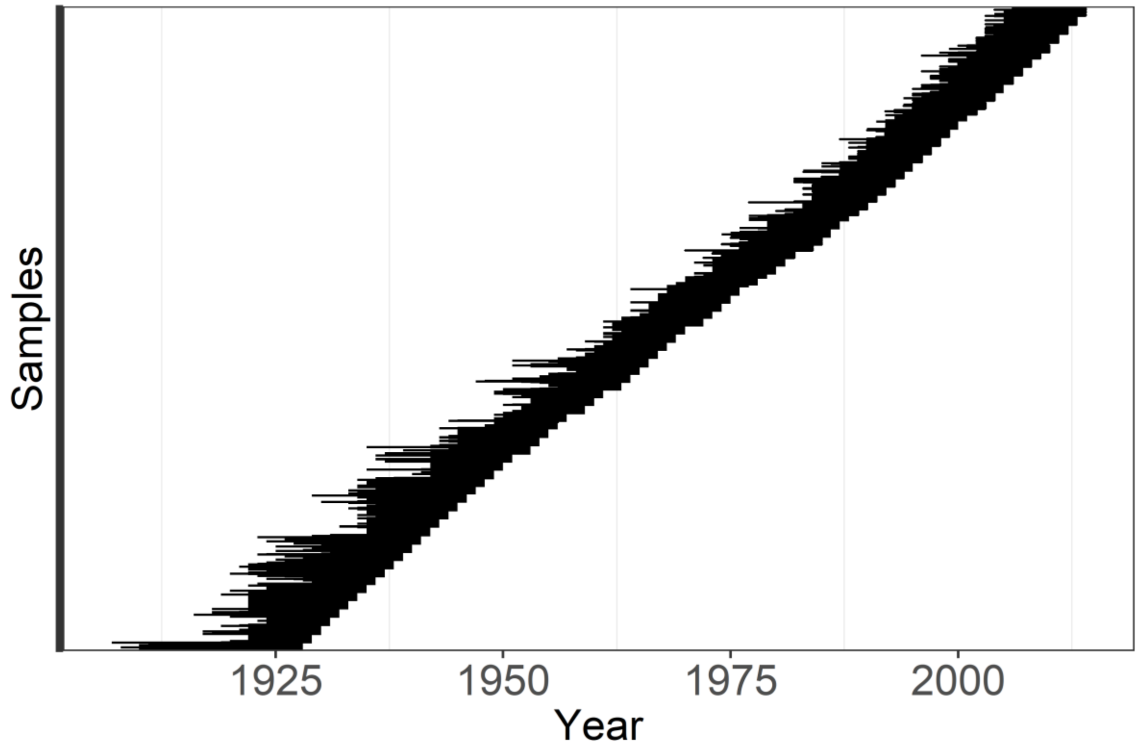

Fig. S3. Sample temporal ranges. Each line represents one sample (otolith) used in the analysis.

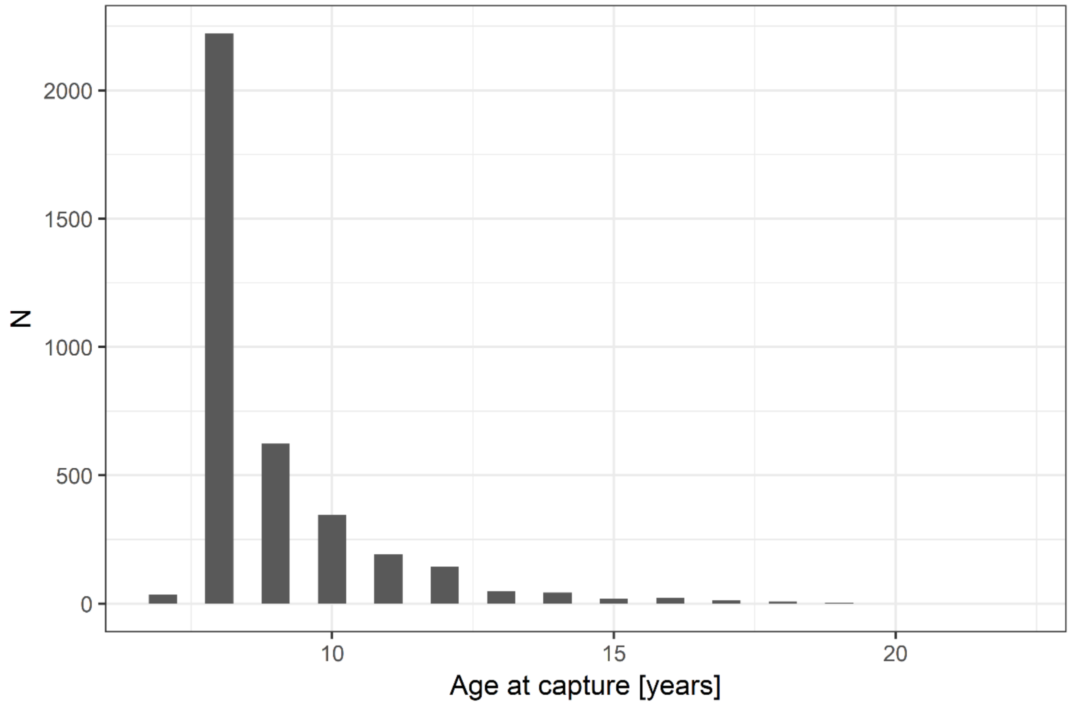

Fig. S4. Age-at-capture frequency of samples.

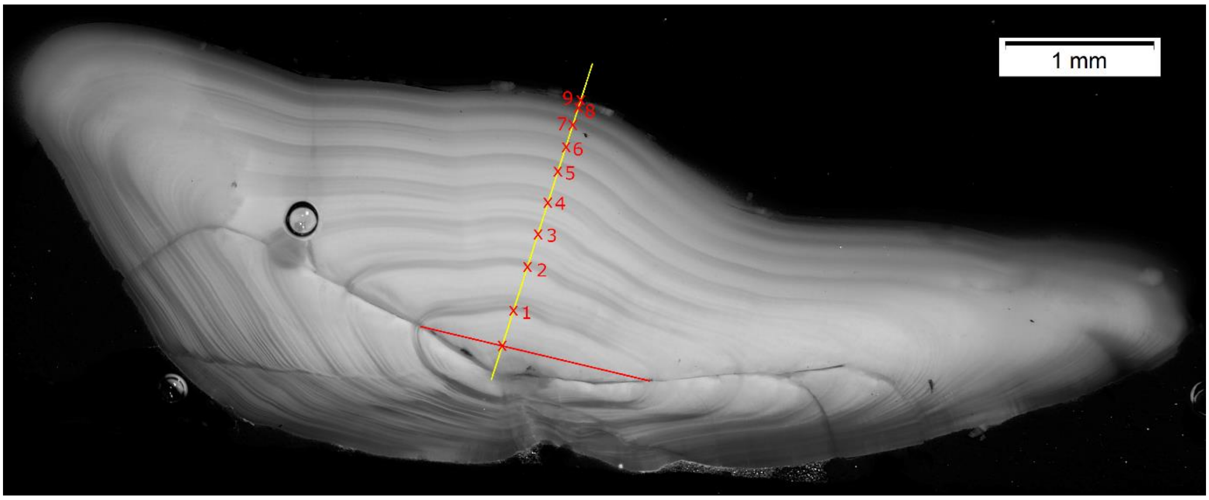

Fig. S5. Atlantic cod (*Gadus morhua*) otolith viewed under reflected light. The measurement axis (yellow line) and annual rings (red crosses) are shown. The last and first increment measurements were excluded from the analysis because they showed incomplete growth.

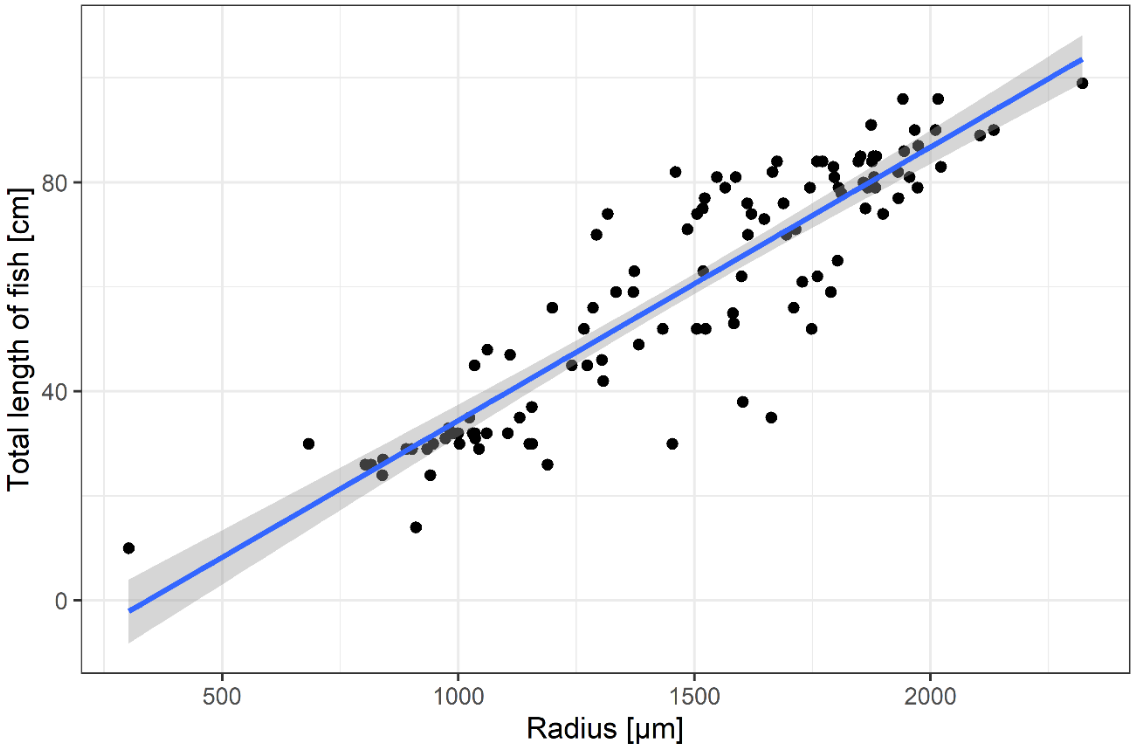

Fig. S6. Relationship between radius measured along the measurement axis on the otolith section and fish total length (Total length~-17.91+0.05*radius, adjusted R^2=0.80, F-statistic: 446.3 on 1 and 109 DF, p-value: < 0.001).

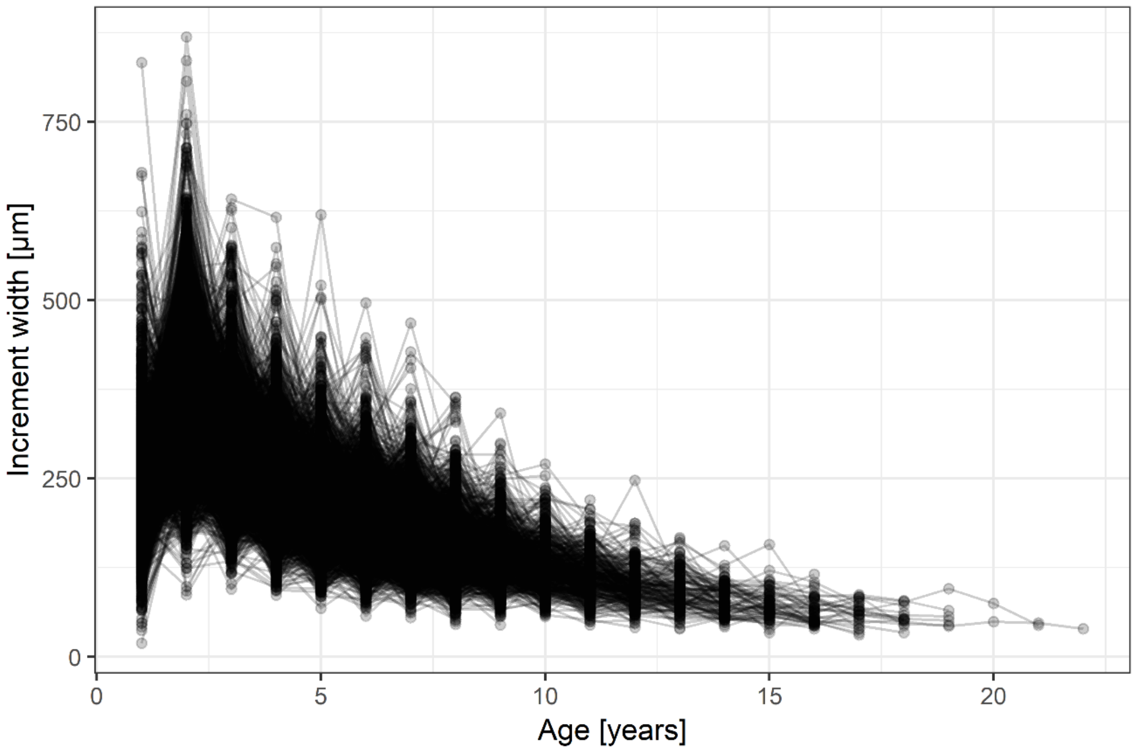

Fig. S7. Individual growth trajectories. The first and last increment measurements were excluded from the analysis because they showed incomplete growth.

Note 1: Stock dynamics and environmental data

The reconstruction of the stock dynamics of the Icelandic cod stock was based on combining the catch at age (age 3-14) matrix for years 1928-1954 (Schopka 1994) and 1955-2017 (ICES 2019). Tuning indices were based on age groups 1 to 10 from the Icelandic spring groundfish survey and Icelandic autumn groundfish survey (NWWG 2019).

The assessment model used was a statistical catch-at-age model with constant selectivity assumption for 6 periods (years 1928-1937, 1938-1949, 1950-1975, 1976-1993, 1994-2003, 2004-2017). Immigration was estimated for the following years and ages: 1930-8, 1933-9, 1953-8, 1958-9, 1959-9, 1960-10, 1962-9, 1964-10, 1969-8, 1970-8, 1972-9, 1980-7, 1981-8, 1990-6 and 2009-6. The natural mortality was scaled to 0.2 for all age groups, the catch weights at age were used to estimate the reference biomass of ages 4 and above and the survey weights and maturity at age from the spring survey were used to estimate the spawning stock biomass. Prior to 1985 spawning weights were based on a regression of the survey and catch weights for the period after 1985.

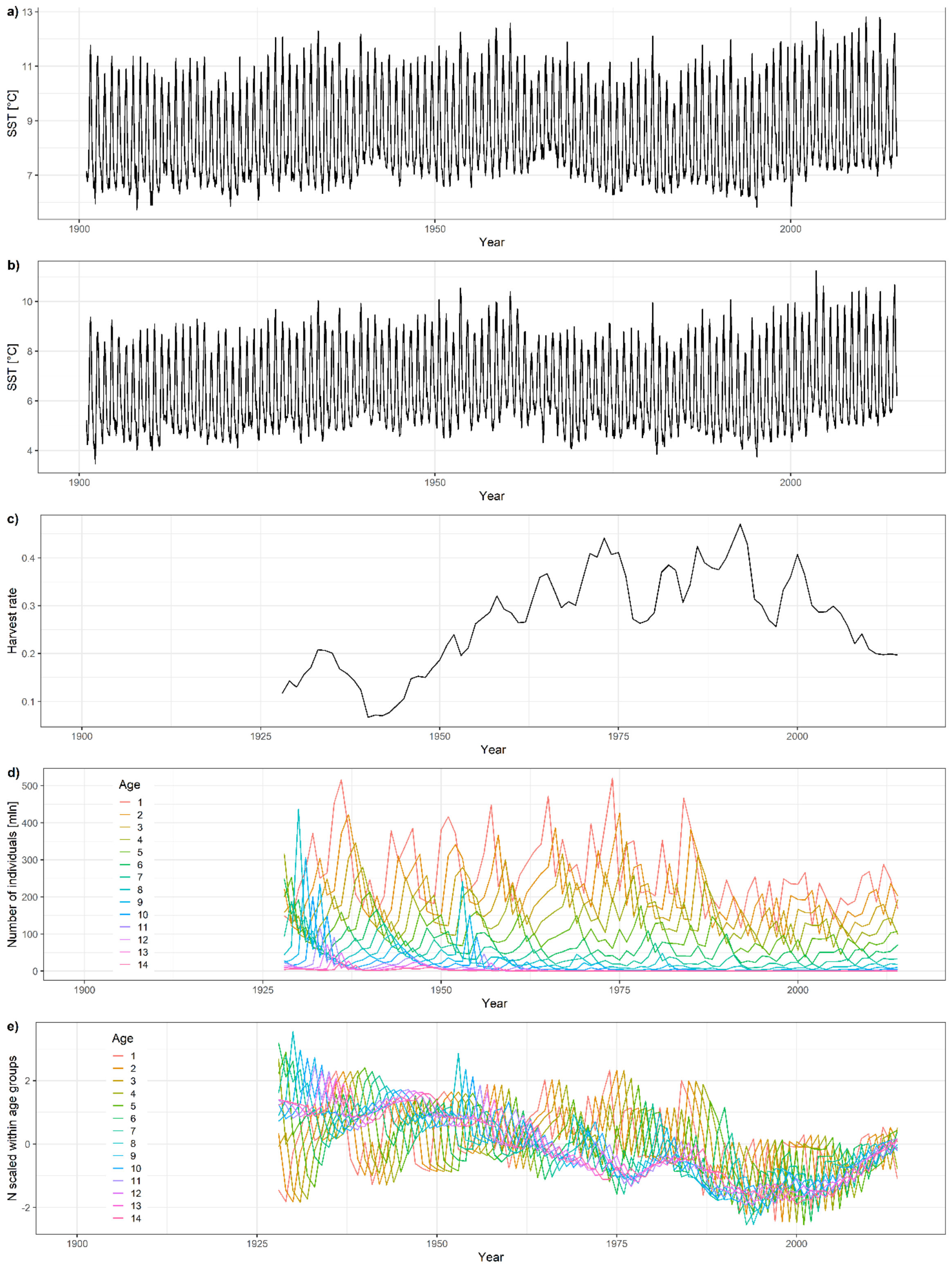

Fig. S8. Environmental, fishing and stock dynamics data used in the study. Mean sea surface temperature in the spawning area (a); mean sea surface temperature in the Icelandic shelf (b); harvest rate for the stock (c); number of individuals at age groups (d); number of individuals scaled within age groups (e).

References:

ICES. 2019. North-Western Working Group. ICES Scientific Reports. 1:14. 620 pp. http://doi.org/10.17895/ices.pub.5298.

Schopka, S. A. (1994). Fluctuations in the cod stock off Iceland during the twentieth century in the fisheries and environment. ICES Marine Science Symposia, 198, 175–193

van de Pol, M., and J. Wright. 2009. A simple method for distinguishing within- versus between-subject effects using mixed models. Animal Behaviour 77:753–758.
